# Post-Thyroidectomy Complications and Risk Factors in Tabuk, Saudi Arabia: A Retrospective Cohort Study

**DOI:** 10.7759/cureus.10852

**Published:** 2020-10-08

**Authors:** Saad M Alqahtani, Basem Almussallam, Amani Salem Alatawi, Nada Awad Alsuhaimi, Amani Albalawi, Nada Saleh Albalawi, Attiya M Alzahrani, Yousef Alalawi

**Affiliations:** 1 Department of Surgery, College of Medicine, Majmaah University, Majmaah, SAU; 2 Department of Surgery, McMaster University, Hamilton, CAN; 3 Department of Surgery, King Fahad Specialist Hospital, Tabuk, SAU; 4 Department of Family Medicine, King Salman Armed Forces Hospital in North-Western Region, Tabuk, SAU; 5 Department of Radiology, King Fahad Medical City, Riyadh, SAU; 6 Department of Ophthalmology, King Fahad University Hospital, Khobar, SAU; 7 Department of Surgery, King Salman Armed Forces Hospital in North-Western Region, Tabuk, SAU

**Keywords:** thyroidectomy, complications, risk factors, hypocalcaemia, voice changes

## Abstract

Background

Thyroid surgery is one of the most commonly performed procedures internationally. There were no studies conducted in Tabuk, Saudi Arabia, on post-thyroidectomy complications and their risk factors.

Objective

The aim of this study was to assess post-thyroidectomy complications and determine the risk factors of such complications.

Methods

This retrospective study included all cases that underwent thyroidectomy at King Salman Armed Forces Hospital, Tabuk, Saudi Arabia, from January 2012 to December 2017. Patients with preoperative hypoparathyroidism, chronic kidney disease, or history of dysphonia were excluded. Data were collected from medical records.

Results

The study showed 182 patients who underwent thyroidectomy operation between January 2012 and December 2017. Temporary hypocalcemia was developed in 116 patients (63.7%) while it persisted in three (1.6%). Change of voice was reported in five patients (2.7%) while two (1.1%) lost a high-pitched voice. Seroma, hematoma, and tracheal injury were documented in 1.6%, 1.1%, and 0.5%, respectively. Multivariate analysis showed that total thyroidectomy was the most significant (four times) risk factor for the development of hypocalcemia as compared to other surgical procedures.

Conclusion

Hypocalcemia was the most frequent post-thyroidectomy complication, whereas voice changes, seroma, hematoma, and tracheal injury are rare complications. Additionally, total thyroidectomy has the highest risk of postoperative hypocalcemia.

## Introduction

Thyroid diseases are considered amongst one of the common endocrine gland disorders worldwide. In the United States alone, an estimated 20 million Americans have some form of thyroid disease [1]. In 2017, 45,379 new cases of thyroid cancer were reported and 1,892 died of thyroid cancer in the United States [2]. The management of thyroid diseases can include medical and/or surgical treatment. Thyroidectomy is one of the most commonly performed procedures in the world and could be either partial or total [3-4].

Nowadays, with the improvement in diagnostic modalities and surgical procedure safety, thyroidectomy surgery has become more feasible. In addition, access to healthcare services has become easier. The surgery indications were also expanded. There are many indications for thyroidectomy, including wandering goiter, compression symptoms, malignancy, or suspected malignancy [3,5].

With newer medical developments and increased surgical experience, thyroidectomy has become a safe procedure with low postoperative morbidity and mortality rates for experienced surgeons with higher annual thyroidectomy volume [6]. However, serious post-thyroidectomy complications do occur, and they include recurrent laryngeal nerve injury, permanent hypoparathyroidism, postoperative bleeding, and hypocalcemia [7-8]. Previous research has investigated the risk factors for thyroidectomy complications. Several factors have been identified, including age, gender, type of thyroid disease, lymph node dissection, and weight of the thyroid gland [9-11].

To the best of our knowledge and on reviewing the current medical literature, there are no studies conducted in Tabuk, Saudi Arabia, about the risk factors and complications of thyroidectomy. Therefore, the aim of this study is to determine the incidence of post-thyroidectomy complications and their risk factors in Tabuk, Saudi Arabia.

## Materials and methods

Methods

We conducted a retrospective cohort study looking at the incidence of post-thyroidectomy complications and associated risk factors. Our study population included all patients who had a thyroidectomy operation performed at King Salman Armed Forces Hospital, Tabuk, Saudi Arabia. We reviewed the medical records for a six-year period between January 2012 and December 2017. A total of 182 cases of thyroidectomies were identified and reviewed. Out of 182 cases, 42 were performed by general surgeons while 140 were performed by endocrine surgeons. We excluded patients with preoperative hypoparathyroidism, chronic kidney disease, and/or a history of dysphonia.

Data extraction was carried out from the patients’ medical records manually. Data were collected to define the risk factors for post-thyroidectomy complications, including age, gender, type of thyroid disease, and type of procedure, and to report post-thyroidectomy complications such as recurrent laryngeal nerve injury, hypocalcemia, hematoma formation, wound infection, hoarseness, loss of high pitch sound, seroma, tracheal injury, and thoracic duct injury. Those complications were established based on clinical assessment aided by biochemical and/or radiological investigations. No routine preoperative parathyroid hormone (PTH) level is measured. Calcium level is measured routinely preoperatively and postoperatively (in both total and subtotal thyroidectomies) every eight hours till the patient is discharged home. Albumin and ionized calcium are measured when calcium is low. Additionally, the PTH level is ordered in case of inappropriate response to calcium replacement. The final pathology result is also checked. Vocal cord assessment is not done routinely preoperatively unless there is clinical concern or before redo surgeries. Postoperatively, vocal cord assessment is done if there is a change in voice or a clinical concern for vocal cord injury. A surgical drain was not placed routinely except in the case of a vascular gland or a huge/large thyroid gland.

 

Statistical analysis

All data were analyzed using SPSS version 22 (IBM Corp., Armonk, NY). Categorical data were presented as numbers and percentages and were analyzed using the chi-square and Fisher’s exact tests. Continuous data were tested for normality by the Shapiro Wilk test. Normally distributed data were expressed as mean ± standard deviation, and they were analyzed with one-way analysis of variance (ANOVA). The risk of hypocalcemia was analyzed using multivariate binary logistic regression analysis, where hypocalcemia was considered as present or not and the type of surgical procedure was classified as total thyroidectomy or not. P-value <0.05 was considered statistically significant.

Ethical considerations

Ethical approval for this study was obtained from the Institutional Research Ethics Committee of King Salman Armed Forces Hospital prior to the conduction of the study. All procedures involving human participants were performed in accordance with the ethical standard of the institutional and/or national research committee, the 1964 Helsinki declaration and its later amendments, and comparable ethical standards. A de-identification coding system was used to protect patient information.

## Results

This study reviewed 182 patients who had undergone thyroidectomy operations between January 2012 and December 2017. Females outnumbered males (83.0% versus 17.0%, respectively). Age ranged from 15.0 to 95.0 years, with a mean age of 39.87±12.67. In 57.7% of the patients, the diagnosis was benign. Regarding the type of procedure, the majority (58.8%) of the patients had a total thyroidectomy, whereas right and left hemithyroidectomy was performed in 21.4% and 13.2% patients, respectively. Subtotal and completion thyroidectomy were done in 3.8% and 2.7%, respectively (Table 1).

**Table 1:** Demographic and operative characteristics of the studied patients (N=182)

Age (years)	Minimum	15.0	
	Maximum	95.0	
	Mean± SD	39.87±12.67	
		N	%
Gender	Female	151	83.0%
	Male	31	17.0%
Type of thyroid disease	Benign	105	57.7%
	Malignant	77	42.3%
Type of surgery	Completion thyroidectomy	5	2.7%
	Right hemithyroidectomy	39	21.4%
	Left hemithyroidectomy	24	13.2%
	Sub-total thyroidectomy	7	3.8%
	Total thyroidectomy	107	58.8%

The frequency of post-thyroidectomy complications was measured from medical charts (Table 2). Hypocalcemia was the most frequent complication; 116 (63.7%) developed temporary hypocalcemia while it persisted in three (1.6%) patients. Change of voice was recorded in five (2.7%) while two (1.1%) lost a high-pitched voice. Seroma, hematoma, and tracheal injury were reported in 1.6%, 1.1%, and 0.5%, respectively. However, there were no reported thoracic duct injury, stridor, or wound infection in our data.

**Table 2:** Frequency of post-thyroidectomy complications

Complication		N=182	%
Change of voice	No	177	97.3%
	Yes	5	2.7%
Stridor	No	182	100%
	Temporary	0	0.0%
	Permanent	0	0%
Loss of high-pitched voice	No	180	98.9%
	Yes	2	1.1%
Hypocalcemia	NO	63	34.6%
	Temporary	116	63.7%
	Permanent	3	1.6%
Hematoma	No	180	98.9%
	Yes	2	1.1%
Seroma	No	179	98.4%
	Yes	3	1.6%
Tracheal injury	No	181	99.5%
	Yes	1	0.5%
Thoracic duct injury	No	182	100.0%
	Yes	0	0.0%
Wound infection	No	182	100.0%
	Yes	0	0.0%

Table 3 shows the association between different risk factors and post-thyroidectomy hypocalcemia. We found a significant statistical association between having thyroidectomy surgery and the development of hypocalcemia (p-value <0.05). Temporary hypocalcemia was significantly higher among patients who underwent total, right hemithyroidectomy and left hemithyroidectomy (70.7%, 15.5%, and 10.3%, respectively). Permanent hypocalcemia was seen only with left hemithyroidectomy, subtotal, and total thyroidectomy. Alternatively, there was no significant association between each of age, gender, or type of thyroid disease and the occurrence of hypocalcemia (p-value>0.05).

**Table 3:** Associations of patient’s demographics, type of thyroid disease, and type of surgery and the risk of hypocalcemia *significant at p<0.05

			Hypocalcemia			
			NO N=63	Temporary N=116	Permanent N=3	P value
Age	Mean		38.62	40.50	41.67	0.621
	SD		11.46	13.41	5.69	
Gender	Female	N	50	98	3	0.67
		%	79.4%	84.5%	100.0%	
	Male	N	13	18	0	
		%	20.6%	15.5%	0.0%	
Type of thyroid disease	Benign	N	38	65	2	0.84
		%	60.3%	56.0%	66.7%	
	Malignant	N	25	51	1	
		%	39.7%	44.0%	33.3%	
Type of surgery	Completion thyroidectomy	N	3	2	0	<0.001*
		%	4.8%	1.7%	0.0%	
	Hemithyroidectomy Right	N	21	18	0	
		%	33.3%	15.5%	0.0%	
	Hemithyroidectomy Left	N	11	12	1	
		%	17.5%	10.3%	33.3%	
	Sub-total thyroidectomy	N	4	2	1	
		%	6.3%	1.7%	33.3%	
	Total thyroidectomy	N	24	82	1	
		%	38.1%	70.7%	33.3%	

Table 4 shows the absence of a significant statistical association between each of age, gender, the type of thyroid disease or the type of surgical procedure, and the incidence of voice changes or loss of high-pitched voice (p-value>0.05).

**Table 4:** Associations of patient’s demographics, type of thyroid disease, and type of surgery and the risk of changes of voice

			Change of voice			Loss of high- pitched voice		
			No N=177	Yes N=5	P value	No N=180	Yes N=2	P value
Age	Mean		39.79	42.80	0.60	39.92	35.00	0.59
	SD		12.69	12.70		12.69	12.73	
Gender	Female	N	146	5	0.59	149	2	1.0
		%	82.5%	100.0%		82.8%	100.0%	
	Male	N	31	0		31	0	
		%	17.5%	0.0%		17.2%	0.0%	
Type of thyroid disease	Benign	N	101	4	0.39	105	0	0.19
		%	57.1%	80.0%		58.3%	0.0%	
	Malignant	N	76	1		75	2	
		%	42.9%	20.0%		41.7%	100%	
Type of surgery	Completion thyroidectomy	N	5	0	1.0	5	0	0.87
		%	2.8%	0.0%		2.8%	0.0%	
	Right hemithyroidectomy	N	38	1		38	1	
		%	21.5%	20.0%		21.1%	50.0%	
	Left hemithyroidectomy	N	24	0		24	0	
		%	13.6%	0.0%		13.3%	0.0%	
	Sub-total thyroidectomy	N	7	0		7	0	
		%	4.0%	0.0%		3.9%	0.0%	
	Total thyroidectomy	N	103	4		106	1	
		%	58.2%	80.0%		58.9%	50.0%	

The development of hematoma showed a significant statistical association with having thyroidectomy surgery (p-value<0.05) (Table 5). Two cases of postoperative hematoma were reported: one in completion thyroidectomy and another in right hemithyroidectomy operation. Conversely, age, gender, and type of thyroid disease did not show an association with hematoma formation as shown in Table 5.

**Table 5:** Associations of patient’s demographics, type of thyroid disease, and type of surgery and the risk of hematoma formation

			Hematoma		
			No N=180	Yes N=2	P value
Age	Mean		39.93	34.50	0.55
	SD		12.72	4.95	
Gender	Female	N	149	2	1.00
		%	82.8%	100.0%	
	Male	N	31	0	
		%	17.2%	0.0%	
Type of thyroid disease	Benign	N	104	1	1.00
		%	57.8%	50.0%	
	Malignant	N	76	1	
		%	42.2%	50.0%	
Type of surgery	Completion thyroidectomy	N	4	1	0.033*
		%	2.2%	50.0%	
	Right Hemithyroidectomy	N	38	1	
		%	21.1%	50.0%	
	Left Hemithyroidectomy	N	24	0	
		%	13.3%	0.0%	
	Sub-total thyroidectomy	N	7	0	
		%	3.9%	0.0%	
	Total thyroidectomy	N	107	0	
		%	59.4%	0.0%	

Seroma formation and tracheal injury did not show any significant relationship to age, gender, type of the disease, or type of surgical procedure (p-value >0.05).

Logistic regression was performed to ascertain the effects of age, gender, type of thyroid disease, and type of surgery on the likelihood that the patient has hypocalcemia. The logistic model was statistically significant (x2=20.69, p-value<0.001*). The model explained 15% of the variance in hypocalcemia and correctly classified 71.4% of cases. Age, gender, type of thyroid disease did not add significantly to the model (p-value>0.05) while the type of surgery, whether total thyroidectomy or not added significantly to the model (p-value<0.001). Total thyroidectomy was 4.09 times more likely to exhibit hypocalcemia in comparison with other types of surgical procedures as illustrated in Table 6.

**Table 6:** Binary logistic regression model for the prediction of hypocalcemia *significant at p<0.05

Regression model	Hypocalcemia	
	P-value	Odds ratio
Age by year	0.16	1.02
Gender	0.12	2.04
Type of thyroid disease	0.69	1.15
Total thyroidectomy	<0.001*	4.09

## Discussion

Postoperative hypocalcemia is commonly seen following a total or subtotal thyroidectomy, with an estimated incidence from 15% to 50% [12]. Indeed, in most cases of hypocalcemia, they resolve spontaneously. However, it may become permanent, requiring lifelong treatment and follow-up. Intravenous therapy may be required, especially in severe cases, and thus prolongs the duration of hospital stay and, substantially, increases the overall cost [13].

Our study showed that post-thyroidectomy temporary hypocalcemia was the most frequent (63.7%) complication while the persistent type was less frequent (1.6%). Having thyroidectomy operations was significantly associated with the risk of developing hypocalcemia with a p-value of <0.001. The total thyroidectomy operation in the current study displayed the highest increase in the risk of developing hypocalcemia in comparison with other surgical procedures with an odds ratio of 4.09. Hypocalcemia is known as one of the most common and serious complications of total thyroidectomy. A multicenter study in Italy found that 10% of patients developed symptomatic hypocalcemia, which was predominantly seen after total thyroidectomy [14]. A recent prospective multicenter study reported a 64.2% total incidence of hypocalcemia after total thyroidectomy. In 49.3% of the participating patients, hypocalcemia was asymptomatic while manifestations of the decreased serum calcium were recorded in only 15% [15]. Furthermore, Pfleiderer et al. reported a 43% incidence of temporary hypocalcemia while 5% developed permanent hypocalcemia that required calcium and vitamin D supplementation for six months postoperative [16]. The risk of hypocalcemia was more frequent after total thyroidectomy as compared to completion thyroidectomy. Moreover, they concluded that patients with toxic goiters and those having a one-stage total thyroidectomy are more likely to experience hypocalcemia.

In our study, both univariate and multivariate analysis demonstrated that neither gender nor age had a significant effect on the occurrence of hypocalcemia. Nonetheless, in the multivariate analysis reported by Eismontas et al., female gender and older age were statistically significant independent predictors of hypocalcemia [15]. Furthermore, Hallgrimsson et al. reported more prevalence of transient postoperative hypocalcemia in younger patients [17]. Kamer et al. found that the risk of hypocalcemia was more frequent in older patients [18].

Recurrent laryngeal nerve (RLN) injury is one of the most feared complications of thyroid surgery. The incidence of RLN injury after thyroidectomy widely varies, ranging from 4% to 7% for temporary paresis and from 1% to 4% for permanent paralysis [19]. Unilateral RLN paralysis commonly manifests clinically as hoarseness, weakness, and breathiness of the voice [20]. Joliat et al. reported a 14% incidence of vocal cord paresis after thyroid surgery [21]. In 1.1% of the entire participants, the injury was permanent. Our data showed that a change of voice was recorded in five (2.7%) of the studied cohort while two (1.1%) of patients in our study have lost their high-pitched voice. Ischemia, entrapment, contusion, irritation of the nerve without actual damage, and actual transection are different mechanisms for RLN injury. Variations and distortions of anatomy are contributing factors that increase the risk of nerve injury [22].

The present study showed an incidence of seroma in 1.6% of patients while hematoma was seen in 1.1%. Our results were within the range that was recorded in the literature of 1% to 2.5% and 0% to 3%, respectively [23]. Our data show that the development of hematoma was significantly associated with having a thyroidectomy operation (p-value<0.05). Two cases of postoperative hematoma were reported: one in completion thyroidectomy and another in right hemithyroidectomy operation.

Our study was a single-center study and was limited by the small number of available cases, the missing additional demographic information, and the retrospective nature of the study.

## Conclusions

In conclusion, hypocalcemia was the most frequent post-thyroidectomy complication, whereas voice changes, seroma, hematoma, and tracheal injury are rare complications. Additionally, total thyroidectomy operation has the highest risk of postoperative hypocalcemia.
